# Prevalence and Perspectives of Use of Dietary Supplements Among Adult Athletes Visiting Fitness Centers in Saudi Arabia

**DOI:** 10.3390/jcm14207410

**Published:** 2025-10-20

**Authors:** Haya I. Aljohar, Hajar F. Almusharraf, Samiah Alhabardi

**Affiliations:** 1 Department of Pharmaceutical Chemistry, College of Pharmacy, King Saud University, P.O. Box 2457, Riyadh 11451, Saudi Arabia; 2 Pharmaceutics Department, College of Pharmacy, King Saud University, Riyadh 11451, Saudi Arabia

**Keywords:** dietary supplements, adults athletes, prevalence, perspective, Saudi Arabia

## Abstract

**Background/Objectives**: Dietary supplement (DS) use has become increasingly prevalent among adult athletes worldwide and carries both potential benefits and risks. This study aimed to examine the prevalence and perspectives of DS use among adult athletes attending fitness centers in Saudi Arabia. **Methods**: A cross-sectional study was carried out between November and December 2024 using self-administered online questionnaires distributed through a convenience sampling method utilizing social media platforms. All adult athletes aged 18 and above currently residing in Saudi Arabia were included. **Results**: Results indicate that 73.9% of athletes use DS, primarily to address self-reported perceived vitamin/mineral deficiencies (62.0%) and to enhance energy availability (45.8%). The most frequently reported DS used by the responded were vitamins and minerals (77.3%), followed by fish oils (57.3%) and proteins (42.7%). The predominant adverse effects reported were changes in urine color (27.4%) and frequent urination (18.0%). Most supplements were consumed orally (78.4%), once daily (40.7%), or according to individual needs (26.7%), with dosage most often determined by a physician or pharmacist (35.1%). The mean perception score of DS effects on health outcomes was 3.69 ± 0.98, with a significant association with age (*p* = 0.041). **Conclusions**: This study highlights the increasing reliance on DS among athletes in Saudi Arabia, highlighting the need for public health interventions that promote safe and informed use of DS. While our study’s use of convenience sampling may limit their generalizability, the findings still provide important insights into current practices and perceptions. Future research should focus on regulatory measures and educational campaigns to mitigate risks and optimize benefits. Our results have significant implications for public health policy and practice.

## 1. Introduction

In recent years, gyms and sports clubs have become very popular for exercising and maintaining overall physical health [1,2]. According to the available literature, there are approximately 1280 health/fitness clubs (gyms) throughout Saudi Arabia (SA) which is highest number in the entire Middle Eastern region, highlighting the need for safe and effective supplements among athletes [3]. Athletes are increasingly resorting to dietary supplements (DS) globally due to a desire to enhance performance, which requires substantial energy expenditure; improve recovery; or achieve specific body composition goals, such as increased muscle mass or weight loss [1,2,4,5,6]. Many athletes are using supplements such as multivitamins, minerals, proteins, herbal supplements, and performance enhancers [4,5]. Unsupervised or excessive use of DS can cause adverse effects. Unlisted ingredients, like anabolic steroids or stimulants, can also cause severe adverse effects, including liver and damage to other organs [7]. However, the safety, effectiveness, and regulation of many DS are still in question, especially when they are used without professional guidance or are at risk of being adulterated [8]. Inadvertent doping, the unintentional use of prohibited substances, is primarily caused by carelessness and DS misuse [9]. Fraudulent practices in the supplement industry further exacerbate these risks, as studies have identified instances where labels do not accurately reflect content [5]. Stimulants (e.g., ephedrine) and anabolic agents are sometimes added to enhance performance, exposing athletes to potential health risks, including liver damage and hormonal imbalances [10,11,12]. Consequently, regulatory bodies, like the World Anti-Doping Agency (WADA), have expressed concerns about banned substances, underscoring the importance of better oversight and education for athletes and their support teams [1].

The global patterns of DS use among athletes vary and show a general trend of increasing consumption, often driven by a heightened awareness of the benefits that supplements can offer in terms of health and well-being [13]. A global scoping review found that the prevalence of regular DS use among athletes ranges from 40% to 90% [14]. For instance, a 2021 study in the United States, reported a 79% prevalence of DS use among both male and female athletes [15]. However, a study found that the prevalence reached 45% among elite collegiate runners from 2 NCAA Division I cross-country teams. Similar trends were observed in countries such as Spain, Australia, and the United Kingdom, with athletes generally showing higher usage rates [16,17].

Within the Middle East, the prevalence of DS use is increasing. Regional studies support these findings. A cross-sectional study conducted in four large indoor fitness centers in Riyadh, Saudi Arabia, reported that 44% of athletes used DS [18]. In Al-ahsa, 60% of individuals who were regularly attended the gym and exercised were using DS [19]. Another study revealed that 44% of adult males registered at fitness centers used DS [20]. Similarly, in another recent study among Saudi women who visited the gym, 68.7% consumed DS [21]. In the western region of Saudi Arabia 52.5% of the athletes consistently used DS [22]. Furthermore, a cross-sectional prevalence study conducted in sports clubs across six Saudi cities, including Al Madinah Al Munawarah, Jeddah, Abha, Tabuk, Dammam, and Riyadh, found that 33.9% of participants used supplements [1]. These findings highlight the increasing reliance on supplements to address perceived nutritional gaps and health concerns.

The Food and Drug Administration (FDA) emphasizes that access to DS should be supervised by healthcare professionals to reduce the risk of misuse, overuse, or interactions with other medications [1,8,13]. Unfortunately, many athletes seek advice from fellow athletes or self-prescribe instead of consulting medical professionals [10]. Further, the FDA has raised serious concerns about certain bodybuilding supplements illegally contain steroids or steroid-like substances, which can cause liver damage [10,23]. The Saudi Arabian Anti-Doping Committee (SAADC) plays a crucial role in ensuring that athletes receive the highest standard of nutrition, doping, and anti-doping education. With the increasing demand for supplements, it is crucial to establish stringent regulatory measures [1].

Despite the increasing consumption of DS, there is limited research on the prevalence, patterns, and perceptions of DS use among adult athletes in Saudi Arabia. As the use of supplements rises, particularly among athletes, the potential health risks, such as herb–drug interactions, remain underexplored [1,24]. In addition, the literature suggests that the rapid expansion of gyms, the promotion of women’s sports, and national initiatives, such as Vision 2030, further increase the consumption of DS [1].

Therefore, the purpose of this study is to explore the prevalence and perspectives of DS use among adult athletes visiting fitness centers in Saudi Arabia.

## 2. Methods

### 2.1. Study Design and Setting

A cross-sectional study was conducted among adult athletes living in Saudi Arabia to evaluate patterns and perceptions related to DS use. Data collection took place from 27 November to 31 December 2024. Adult athletes aged 18 years and older, of both genders, currently residing in Saudi Arabia, were eligible to participate. The study also included professional athletes. Professional athletes were defined as individuals formally registered in sports federations or clubs and actively competing at regional, national, or international levels [25,26]. All athletes had been practicing their respective sports for a minimum of two years. Those who were able to read and understand the questionnaire were included in the study, while individuals who did not meet the inclusion criteria were excluded.

### 2.2. Sample Size and Sampling Method

The sample size for this study was calculated similarly to previous studies [24,27,28]. The sample size was computed using the Raosoft sample size calculator at a 95% confidence interval (CI)and a 5% margin of error (ME) [29]. According to the literature, the total population of athletes in Saudi Arabia is 336,739 [30]. Therefore, the required sample size was computed to be 384. However, to avoid sampling bias and missing responses and to further to strengthen the study, a total of 500 athletes were invited to participate through social media platforms. Recruitment continued until this number was reached during the data collection period. We used a convenient sampling method, and the questionnaire was posted on various social media platforms (Twitter, Facebook, Instagram, and WhatsApp Status).

### 2.3. Construction, Validation, and Reliability of Study Tool

The questionnaire was developed based on an extensive review of the existing literature published on this topic [31,32,33,34]. The survey tool consisted of 33 questions across 7 sections designed to collect information on demographic variables, health status, supplement usage patterns, motivations, and perceptions regarding DS. The first section included ten items assessing athlete’s demographic characteristics, such as age, gender, city of residence, nationality, occupation, educational level, and health status, including vitamin and mineral deficiencies. The second section comprised five items about general information on exercise and lifestyle patterns, focused on athletes’ affiliations with gyms or sports clubs, frequency and type of exercise, and motivations for physical activity (e.g., improving health, weight loss, or building muscle). The third section included five items evaluating knowledge of DS by exploring awareness of DS types, benefits, reasons for using or avoiding DS, and reported side effects. Responses were recorded on a binary scale (Yes/No). The fourth section collected detailed information on the frequency and types of supplements used, methods of intake (e.g., oral, or intravenous), and adherence to dosing recommendations. The fifth section contained four items regarding awareness of the sources, safety, and regulations surrounding DS. Responses were recorded on a binary scale (Yes/No). The sixth section addressed athletes’ practices concerning blood tests conducted before and during DS usage. The last section focused on perceptions toward DS (10 items), evaluated using a five-point Likert scale with the following scores: Strongly Agree = 1, Agree = 2, Neutral = 3, Disagree = 4, and Strongly Disagree = 5. These items addressed perceptions of safety, efficacy, and the role of DS in enhancing health and performance. The final bilingual questionnaire (English and Arabic) was disseminated through social media platforms and personal contacts.

After the initial draft of the questionnaire, it was translated into Arabic using a forward and backward translation procedure. The questionnaires were then reviewed by experts, including a researcher and two professors specializing in the fields of pharmaceutical chemistry and epidemiology to ensure content validity of the questionnaire. The feedback from the experts was taken into consideration during the revision process. However, the experts’ opinions indicated that no changes to the questionnaire were necessary. To ensure face validity, the revised questionnaire used in this study was piloted on a randomly selected 20 individuals to ensure that the questionnaires were linguistically and conceptually understood. The results from this pilot study were not included in the final data analysis. Additionally, the reliability of the questionnaires was determined using the Cronbach alpha coefficient, which scored 0.822, indicating good internal consistency [35]. This supports the questionnaire’s reliability, as Cronbach’s alpha is a suitable measure for evaluating reliability, according to previous research [35].

### 2.4. Ethical Consideration

Prior to data collection, the study questionnaires were reviewed and approved by the ethics committee of scientific research at King Saud University (KSU-HE-24-1053), dated 26 November 2024. Informed consent was obtained from all athletes, who were assured that their anonymized data would be used for scientific publication and research purposes only, and that confidentiality would be maintained throughout the study. They also had the right to withdraw from the study at any time. The study procedures were conducted in accordance with the guidelines and regulations of the Declaration of Helsinki for human research.

### 2.5. Statistical Analysis

Data was collected in Excel and then transferred to the Statistical Software Package, version 27 (SPSS Inc., Armonk, NY, USA), for analysis. Descriptive statistics, such as frequency (*n*) and percentage (%), were summarized for categorical variables, while continuous variables were displayed as mean and standard deviation. Independent *t*-tests and one-way ANOVA were utilized to determine differences between the variables. All statistical analyses were conducted at a significance level of 0.05.

## 3. Results

### 3.1. Participant Recruitment and Characteristics

**Figure 1:**
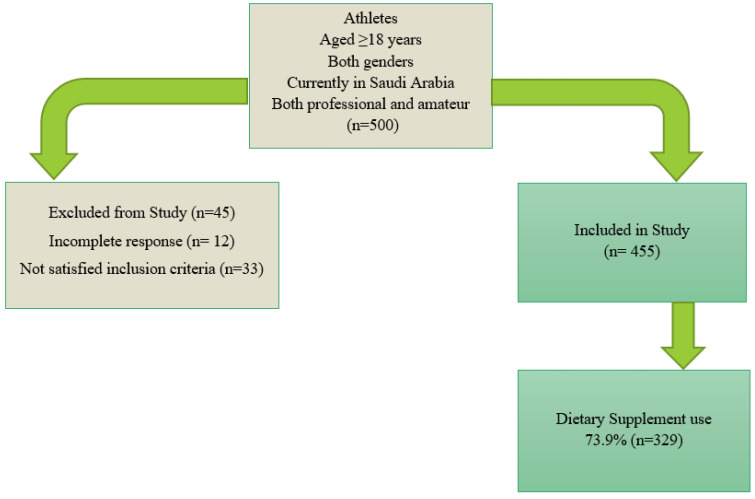
Flow chart of the study.

**Figure 2:**
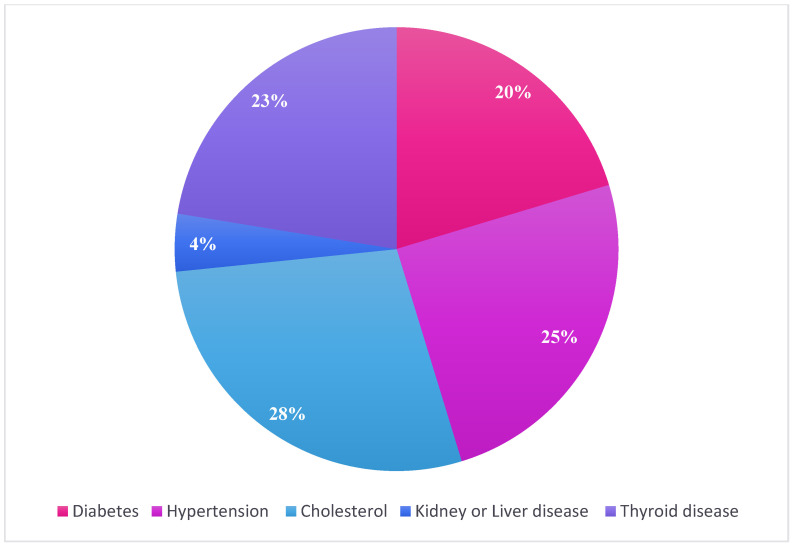
Athletes’ health problems.

A total of 500 participants responded to the survey after providing informed consent. However, 45 were excluded because of incomplete responses (12) and did not meet the inclusion criteria (33). Therefore, 445 responses were included in the final analysis, resulting in a completion rate of 89%. (Figure 1). Most participants were female (383; 86.1%), aged over 46 years (189; 42.5%) and were Saudi nationals residing in Riyadh. In terms of educational level, the majority had a university-level education (67.0%). Table 1 shows that 7.0% of athletes were current smokers, while 90.8% were not, and 2.2% were former smokers. Regarding health conditions, 31.9% reported having health issues, while 68.1% did not. A significant portion (51.9%) reported deficiencies in vitamins and minerals. The distribution of athlete’s health issues is presented in Figure 2.

### 3.2. Sports Club Affiliation and Exercise Patterns

Almost 40% of the participants were affiliated with independent private clubs, 6.5% with governmental entities, and 1.8% with private hospitals. Regarding licensing status, 45.8% reported that their clubs were licensed, 42.0% were unsure, and 12.1% indicated that their clubs were unlicensed. Only 22 athletes answered the question on exercise motivation besides their specific training related to sports. Among them, the most common reasons were building muscle (31.8%), improving health (27.3%), and weight loss (22.7%). The frequency of exercise showed that 33.0% exercised 2–3 times weekly, while 22.5% exercised weekly (Table 2).

**Figure 3:**
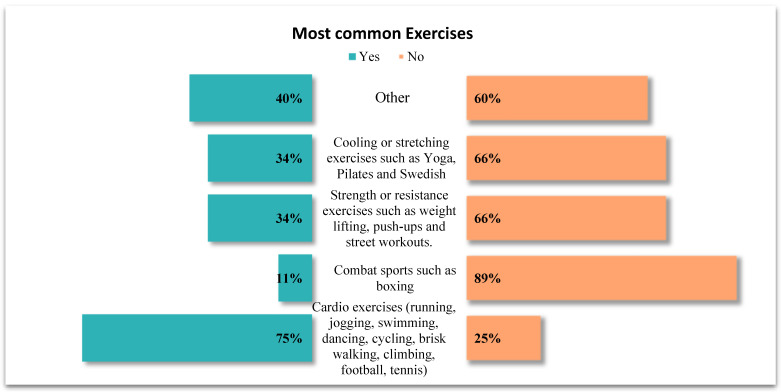
Athletes’ current types of exercise.

Most participants (75.3%) performed cardio exercises, and approximately (34%) engaged in strength or resistance training. The detailed distribution of exercise types is presented in Figure 3.

### 3.3. Dietary Supplement (DS) Use

DS use was highly prevalent, reported by 73.9% of participants. The primary reason was to address vitamin/mineral deficiencies (62%) and enhance energy levels (45.8%). Vitamins and minerals were the most frequently used (77.3%), followed by fish oils (57.3%) and proteins (42.7%). The predominant adverse effects reported were altered urine characteristics (27.4%) and increased frequent urination (18.0%) (Table 3).

Table 4 illustrates detailed information about the frequency of usage of DS. A total of 25.8% reported regular use of DS. The majority (78.4%) used DS orally. Regarding the frequency, 40.7% took supplements once a day. Dosage determination was most commonly guided by a specialist doctor or pharmacist (35.1%). Commitment to supplement use varied, with 33.7% using them temporarily until desired results were achieved. Advice regarding supplement use was most often obtained from medical professionals (41.1%), followed by self-guidance (22.7%) and nutrition specialists (8.5%).

### 3.4. Supplementation Practices and Purchasing

Regarding purchasing behavior, 65.6% bought DS from pharmacies, while 41.8% purchased them online. Most (78.2%) bought products in their original packaging, whereas 2.9% purchased items not in original packaging. For information sources, 57.5% relied on physicians or pharmacists, 39.1% on nutrition specialists, and 30.1% on friends (Table 5).

### 3.5. Blood Testing and Supplement Use

More than half of the participants (57.3%) reported having a blood test before starting supplementation, while 24.3% did not, and 18.4% responded that it was not relevant to them. Among those who underwent testing, 40.9% did not specify any timeframe, while others reported testing annually (20.4%) or every six months (20.0%). Regarding blood testing during supplement use, only 15.3% of athletes had undergone testing, while 47.0% did not (Table 6).

### 3.6. Use of Hormones, Proteins, and Stimulants

**Figure 4:**
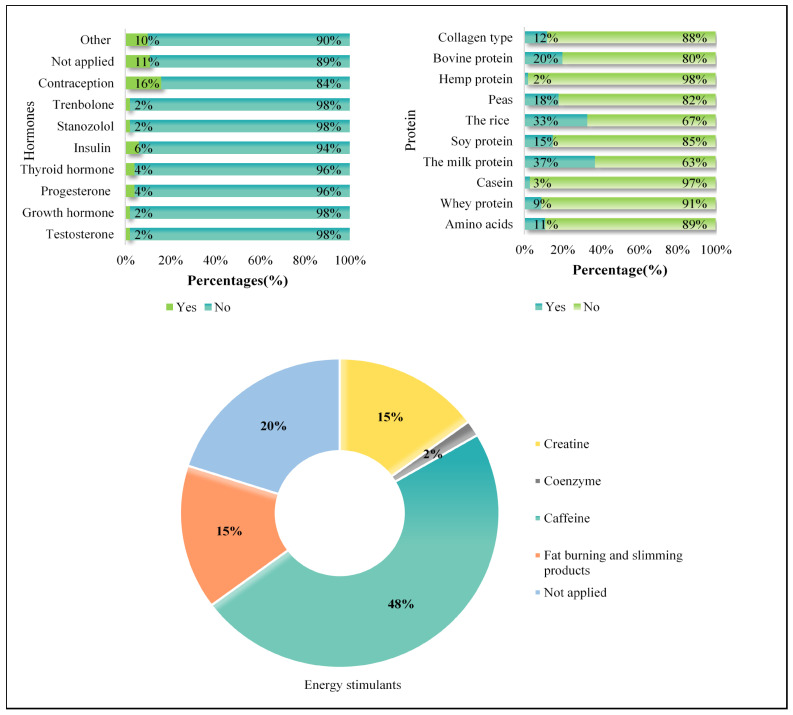
Usage patterns for hormones, proteins, and energy stimulants.

Figure 4 shows the usage patterns of various DS. Among hormones, contraception was the most commonly used (16.4%), followed by insulin (5.8%) and testosterone (2.2%). For protein supplements, milk protein was the most preferred (36.9%), followed by rice protein (32.6%) and bovine protein (19.6%). Caffeine was the leading energy stimulant (26.5%), while creatine and fat-burning products were reported by 8.3%, and coenzyme Q10 by 0.9%.

### 3.7. Perceptions of Supplement Use

The analysis of perception scores indicated that the highest agreement was for the statement “Sports clubs should provide awareness lectures on DS” (mean = 3.85), reflecting a strong demand for education on supplement use. Perceptions of the safety of commercially available supplements in stores and markets were moderate, with a mean score of 3.33, while the statement “Taking supplements replaces the need for a balanced diet” had the lowest agreement (mean = 2.40), indicating awareness of the importance of proper nutrition (Table 7).

The overall mean perception score of DS on the health outcomes was 3.69 ± 0.98. Gender was not significantly associated with perception scores (*p* = 0.245), while age showed a significant association (*p* = 0.041) (Table 8).

## 4. Discussion

The landscape of DS use has significantly transformed in Saudi Arabia, driven by the goal of improving physical health. However, the safety of these products is of critical concern, as unsupervised use can lead to adverse effects. A key issue is the accuracy of the supplement labels; discrepancies may lead to consumer fraud, highlighting fraudulent industry practices [36]. Specifically substances such as stimulants (e.g., ephedrine and pseudoephedrine), anabolic agents, and peptide hormones are often unlisted or misrepresented, leading to harmful consequences when intentionally added to enhance performance [36]. This risk was quantified by a meta-analysis on contamination in DS, which reported that almost 28% of product could result in inadvertent doping, with 875 out of 3132 DS containing undeclared substances [11].

The high prevalence of DS use among Saudi athletes in this study aligns with global trends among physically active adults. Usage rates in countries like the United States, Australia, the United Kingdom (40% to 100%), Spain (96.1%), and Iran (57.9%) demonstrate a common pattern of high consumption, often influenced by global marketing and social media [10,37,38]. A previous study in Saudi Arabia showed a 47.9% prevalence of DS use [39]. The findings of this study highlight that DS use among Saudi athletes is higher than some earlier reports. This increase likely reflects a growing interest in health and wellness, alongside enhanced advertising and greater product availability in pharmacies and online platforms [32,40].

This study found that 28.5% of athletes reported using DS regularly. The most commonly used types of supplements were vitamins and minerals (77.3%), followed by fish oils (57.3%), highlighting key health motivations. These findings align with several regional studies who reported multivitamins, proteins, minerals, and omega-3 fatty acid as the most frequently used DS [1,15,19,41,42,43], whereas a study about the use of hormones and DS among gym attendees reported that 7.9% took hormones and 83.1% consumed protein powder [39]. These consumption patterns are influenced by various factors, including age, gender, education level, and health concerns, with younger adults, those with higher education, and those in the health sector more likely to use supplements [31,44]. Furthermore, cultural norms and dietary practices in Saudi Arabia, where the traditional diet may lack essential nutrients such as vitamin D and iron, make supplementation a necessary strategy for many. The growing fitness culture, driven by national initiatives like Saudi Vision 2030, has increased the demand for supplements aimed at improving physical performance. This shift reflects broader global trends where athletes and fitness enthusiasts increasingly rely on supplements to meet their nutritional needs and optimize their health.

In this study, the primary reasons for using DS were to address deficiencies in vitamins and minerals (62%) and to improve energy and physical activity (45.8%). These findings align with previous research indicating that individuals often use supplements to enhance overall health and performance [1,33,45]. Notably, the current study highlights that athletes in Saudi Arabia are primarily motivated to use DS to enhance physical performance and increase muscle mass, consistent with global trends. Similar motivations were reported in studies by Moradi et al. and AlRuthia et al., where athletes used supplements to improve athletic abilities, stamina, energy, and concentration [18,37]. Regarding adverse effects, 32.6% of athletes reported none, while others experienced changes in urine color or smell (27.4%), frequent urination (18.0%), sleep disturbances (11.0%), and behavioral changes. These findings underscore the potential risks associated with unsupervised DS use.

The findings of this study highlight the widespread use of DS among adult athletes in Saudi Arabia, influenced by factors such as performance enhancement, recovery, and overall health maintenance. In addition, factors such as aggressive marketing, weak regulatory oversight, and a growing fitness culture strongly shaped by aesthetics and body image could be brought to the fore. In addition, the high demand for educational lectures, as captured by perception scores, may be explicitly linked to gaps in health literacy and potential public health interventions [1,39,46]. Furthermore, contextual factors, such as climate and training conditions in Saudi Arabia, like exercising in hot environments, may foster beliefs that supplements (e.g., electrolytes, vitamins) are essential for performance and recovery [47]. Religious practices, like fasting during Ramadan, may also influence supplement use to maintain training and recovery [48].

Emerging studies suggest that psychological and behavioral factors play significant roles in supplementation consumption [49,50]. These findings align with the perspective that athletes may turn to dietary supplements as a compensatory strategy for imbalanced dietary behaviors or underlying psychological predispositions [49]. Understanding these factors is crucial for developing effective education and intervention strategies. Future research should investigate the psychological determinants of DS consumption, including body image concerns, stress, and eating behaviors.

However, the risks associated with DS use are notable, with approximately 23,000 emergency department visits annually attributed to adverse events caused by supplements [51]. Previous studies have also highlighted a reliance on informal sources like coaches, peers, and online platforms for DS information [19,31]. To address this, there is a need for accessible nutritional education and consultation for athletes and coaches. Sports dietitians, nutritionists, and coaches should evaluate DS for legality, safety, and efficacy, and monitor their impact on athlete health and performance [52].

This study has several limitations. Firstly, the reliance on self-reported data introduces potential recall bias, and the cross-sectional design limits causal inference. Secondly, the use of the sampling convenience method and recruitment primarily through social media may have introduced selection bias, skewing the sample toward urban, digitally connected athletes, and underrepresenting those from rural areas. Thirdly, the sample was also predominantly male, which limits the applicability of the findings to female athletes. Furthermore, the study’s findings on exercise motivations are limited by a high rate of non-response, which may introduce bias and affect the representativeness of these findings. Given the sensitivity of certain practices, such as hormone or performance-enhancing substance use, underreporting is possible. These factors constrain the generalizability of the results to the wider athlete population in Saudi Arabia. Future research should aim for more balanced gender representation, employ diversified recruitment strategies beyond social media, and incorporate approaches that allow for more confidential reporting of sensitive behaviors. In addition, examining the efficacy and safety of commonly used DS among athletes and assessing the role of socio-economic factors in influencing DS consumption patterns are required.

## 5. Conclusions

This study highlights the high prevalence of DS use among athletes in Saudi Arabia, with vitamins, minerals, fish oils, and proteins being the most commonly consumed. While many athletes reported using DS to address nutritional deficiencies and enhance energy availability, a notable proportion also experienced adverse effects, underscoring concerns about product safety, labeling accuracy, and misuse. These findings reinforce the urgent need for public health interventions that combine education with stronger regulatory oversight. Aligning such efforts with Saudi Arabia’s Vision 2030—particularly its goals of fostering healthier lifestyles and advancing sports development—can enhance their impact at the national level. Furthermore, situating these initiatives within the framework of international anti-doping standards would strengthen their credibility and ensure that Saudi athletes are safeguarded from both health risks and violations of global sporting regulations. Future research should therefore focus on evaluating the effectiveness of targeted educational campaigns, such as workshops in fitness centers and digital information platforms, alongside regulatory measures aimed at ensuring safe and informed DS use.

## Figures and Tables

**Table 1:** Socio-demographic and clinical characteristics of the athletes (*n* = 445).

Variables	Frequency (%)
Gender	
Male	62 (13.9)
Female	383 (86.1) *
City	
Riyadh	380 (85.4) *
Dammam	6 (1.3)
Jeddah	12 (2.7)
Makah	8 (1.8)
Medina	5 (1.1)
Hail	5 (1.1)
Other	29 (6.5)
Nationality	
Saudi	436 (98.0) *
Non-Saudi	9 (2.0)
Age	
18–24 Years	49 (11.0)
25–35 Years	101 (22.7)
36–40 Years	41 (9.2)
41–45 Years	65 (14.6)
>46 Years and above	189 (42.5) *
Occupation	
Student	45 (10.1)
Government sector employee	142 (31.9)
Military	8 (1.8)
Private sector employee	49 (11.0)
Free business	43 (9.7)
Other	158 (35.5) *
Education Level	
Middle school or less	10 (2.2)
High school	74 (16.6)
University	298 (67.0) *
Postgraduate	63 (14.2)
Health problem	
Yes	142 (31.9)
No	303 (68.1) *
Are you deficient in vitamins and minerals?	
Yes	231 (51.9)
Don’t know	117 (26.3)
No	97 (21.8)
Do you smoke?	
Yes	31 (7.0)
No	404 (90.8) *
Ex. Smoker	10 (2.2)

* Indicates the highest percentage.

**Table 2:** Distribution of athletes: sports club affiliations, licensing awareness, exercise motivations, and frequency patterns (*n* = 445).

Variables	Frequency (%)
Your sports club is affiliated with	
Governmental entity	29 (6.5)
Private hospital	8 (1.8)
Independent private club	177 (39.8)
Other	231 (51.9) *
Does your sports club have a license from the municipality?	
Yes	204 (45.8) *
I don’t know	187 (42.0)
No	54 (12.1)
Besides specific training related to your sport, what are other reasons for your exercise? (*n* = 22 valid response) #	
Lifestyle	1 (4.5)
To improve health	6 (27.3)
To improve body shape	1 (4.5)
To improve health and body shape, and weight loss	1 (4.5)
To build muscle	7 (31.8) *
For weight loss only	5 (22.7)
Hobby	1 (4.5)
Exercise	
Daily	33 (7.4)
4–5 times a week	78 (17.5)
2–3 times a week	147 (33.0) *
Once a week	100 (22.5)
Rarely	87 (19.6)

* Indicates the highest percentage. # Percentages are based on valid responses (*n* = 22). Missing responses (*n* = 423) were excluded.

**Table 3:** General information about dietary supplements.

Variables	Yes *n* (%)	No *n* (%)
Using vitamins or dietary supplements	329 (73.9) *	116 (26.1)
Reasons for use of dietary supplements		
To increase physical strength	151 (33.9)	294 (66.1)
For muscle building and bodybuilding	101 (22.7)	344 (77.3)
Increase activity and energy	204 (45.8)	241 (54.2)
Deficiency in vitamins and minerals	276 (62.0)	169 (38.0)
For weight loss	92 (20.7)	353 (79.3)
Muscle drying	24 (5.4)	421 (94.6)
To reduce injuries	82 (18.4)	363 (81.6)
Not applied	53 (11.9)	392 (88.1)
Other	51 (11.5)	394 (88.5)
Reasons for not using dietary supplements		
I don’t need it	322 (72.4)	123 (27.6)
Unhealthy	78 (17.5)	367 (82.5)
I don’t know much about it	142 (31.9)	303 (68.1)
Expensive	124 (27.9)	321 (72.1)
Other	97 (21.8)	348 (78.2)
Types of dietary supplements		
Vitamins and minerals	344 (77.3)	101 (22.7)
Fish oils (omega-3, omega-6)	255 (57.3)	190 (42.7)
Herbs (ginseng, ginkgo, ephedra)	58 (13.0)	387 (87.0)
Stimulants such as amphetamines	23 (5.2)	422 (94.8)
Energy drinks like Red Bull	28 (6.3)	417 (93.7)
Healthy drinks	149 (33.5)	296 (66.5)
Diuretics such as fursamide, metolazone, and bendrflumethiazide	16 (3.6)	429 (96.4)
Energy and performance stimulants (creatine, caffeine, coenzyme, fat-burning and slimming products)	62 (13.9)	383 (86.1)
Hormones (testosterone, growth hormone, progesterone, thyroid hormone)	31 (7.0)	414 (93.0)
Proteins (amino acids and casein)	69 (15.5)	376 (84.5)
Not applicable	8 (1.8)	23 (5.2)
Side affects you experience while using dietary supplements		
Changes in attitude and behavior, e.g., aggressiveness	18 (4.0)	427 (96.0)
Appearance of acne	41 (9.2)	404 (90.8)
Protrusion in breast	21 (4.7)	424 (95.3)
Increase in height	14 (3.1)	431 (96.9)
Frequent urination	80 (18.0)	365 (82.0)
Oliguria	13 (2.9)	432 (97.1)
Change in the color or smell of urine	122 (27.4)	323 (72.6)
Hair loss	39 (8.8)	406 (91.2)
Noticeable change in sexual activity	43 (9.7)	402 (90.3)
Frequent forgetfulness or changes in memory	42 (9.4)	403 (90.6)
Lack of sleep	49 (11.0)	396 (89.0)
No side effects	145 (32.6)	300 (67.4)
Other	43 (9.7)	402 (90.3)

* Indicates the highest percentage.

**Table 4:** Frequency of usage of dietary supplements.

Variables	Frequency (%)
Regular use of dietary supplements	
Yes	115 (25.8)
No	238 (53.5)
Not applicable	92 (20.7)
Method of Use	
Oral	349 (78.4) *
Intravenous	2 (0.4)
Not applied	90 (20.2)
Other	4 (0.9)
How often do you take daily dietary supplements?	
Once a day	181 (40.7) *
Twice a day	24 (5.4)
Three times a day	8 (1.8)
As needed	119 (26.7)
Not applied	102 (22.9)
Other	11 (2.5)
On what basis was the dietary supplement dose you need determined?	
Based on my body weight	34 (7.6)
By a nutritionist	51 (11.5)
By my athletic trainer	7 (1.6)
By a specialist doctor or pharmacist	156 (35.1) *
I followed the instructions on the product packaging	72 (16.2)
Not applied	119 (26.7)
Other	6 (1.3)
How committed are you to using dietary supplements?	
Continuously throughout the year	83 (18.7)
Temporarily (a week to a month)	79 (17.8)
Temporarily until the desired results are obtained	150 (33.7) *
Not applied	124 (27.9)
Other	9 (1.9)
Whose advice you take on how to use dietary supplements?	
Medical	183 (41.1) *
Sport trainer	14 (3.1)
Nutrition specialist	38 (8.5)
Myself	101 (22.7)
Not applied	103 (23.1)
Other	6 (1.4)

* Indicates the highest percentage.

**Table 5:** Purchasing information about dietary supplements.

Items	Yes *n* (%)	No *n* (%)
Where do you buy dietary supplements?		
Pharmacy	292 (65.6)	153 (34.4)
Dietary supplement stores	104 (23.4)	341 (76.6)
Internet sites	186 (41.8)	259 (58.2)
Sports coach	18 (4.0)	427 (96.0)
Unknown shops	14 (3.1)	431 (96.9)
Not applied	61 (13.7)	384 (86.3)
Other	27 (6.1)	418 (93.9)
Condition of the dietary supplement product when it was purchased		
In the original packaging	348 (78.2)	97 (21.8)
Not in its original packaging	13 (2.9)	432 (97.1)
There is no writing on the packaging indicating its ingredients	24 (5.4)	421 (94.6)
The information on the packaging is incomprehensible	32 (7.2)	413 (92.8)
Not applied	62 (13.9)	383 (86.1)
Other	32 (7.2)	413 (92.8)
Where do you get your information about dietary supplements?		
Sports coach	66 (14.8)	379 (85.2)
A specialist doctor or pharmacist	256 (57.5)	189 (42.5)
Nutrition specialist	174 (39.1)	271 (60.9)
Friends	134 (30.1)	311 (69.9)
Ads	73 (16.4)	372 (83.6)
Not applied	61 (13.7)	384 (86.3)
Other	57 (12.8)	388 (87.2)

**Table 6:** Information about athletes’ practices related to blood tests before and during use of dietary supplements.

Variables	Frequency (%)
Did you take a blood test before taking the supplements?	
Yes	255 (57.3) *
No	108 (24.3)
Not applied	82 (18.4)
The period between each blood test	
Month	21 (4.7)
Three months	52 (11.7)
Six months	89 (20.0)
Every year	91 (20.4)
Not applied	182 (40.9) *
Other	10 (2.2)
Did you take a blood test before taking the supplements?	
Yes	68 (15.3)
No	209 (47.0) *
I haven’t stopped using it	35 (7.9)
Not applied	133 (29.9)

* Indicates the highest percentage.

**Table 7:** Perceptions of dietary supplements on health and physical structure.

Items	Strongly Agree *n* (%)	Agree *n* (%)	Neutral *n* (%)	Disagree *n* (%)	Strongly Disagree *n* (%)	Mean ± SD
DS increase my health and my physical structure	96 (21.6)	176 (39.6)	126 (28.3)	34 (7.6)	13 (2.9)	3.69 ± 0.98
DS available in stores and commercial markets have been previously examined and confirmed to be safe to use	58 (13.0)	137 (30.8)	165 (37.1)	65 (14.6)	20 (4.5)	3.33 ± 1.02
DS provide me with vigor and energy	75 (16.9)	176 (39.6)	140 (31.5)	44 (9.9)	10 (2.2)	3.59 ± 0.96
DS increase my ability to perform exercises	72 (16.2)	165 (37.1)	155 (34.8)	43 (9.7)	10 (2.2)	3.55 ± 0.95
DS increase my concentration	60 (13.5)	153 (34.4)	168 (37.8)	55 (12.4)	9 (2.0)	3.45 ± 0.94
Protein supplementation is essential for a toned body	50 (11.2)	118 (26.5)	187 (42.0)	70 (15.7)	20 (4.5)	3.24 ± 0.99
There is no difference between taking DS with a prescription or without a prescription	23 (5.2)	52 (11.7)	165 (37.1)	157 (35.3)	48 (10.8)	2.65 ± 0.99
Taking food supplements replaces eating enough and varied food	27 (6.1)	37 (8.3)	116 (26.1)	173 (38.9)	92 (20.7)	2.40 ± 1.09
I advise others to take DS	40 (9.0)	112 (25.2)	194 (43.6)	80 (18.0)	19 (4.3)	3.17 ± 0.97
Not taking DS will reduce my athletic performance and physical activity	27 (6.1)	79 (17.8)	181 (40.7)	126 (28.3)	32 (7.2)	2.87 ± 0.99
Sports clubs should provide awareness lectures on DS	152 (34.2)	137 (30.8)	101 (22.7)	49 (11.0)	6 (1.3)	3.85 ± 1.05
The use of DS is a negative phenomenon that must be addressed	33 (7.4)	62 (13.9)	197 (44.3)	114 (25.6)	39 (8.8)	2.86 ± 1.01

DS = Dietary supplements.

**Table 8:** Association between gender and age with mean perception towards dietary supplements increase health and physical structure.

Variables	*n*	Mean	T	F	*p* Value
Gender					
Male	62	37.65 ± 7.67	−1.164		0.245 *
Female	383	38.83 ± 7.36			
Age (years)					
24 Years and below	49	37.51 ± 7.81		2.515	0.041 ^†^
25–35 Years	101	38.94 ± 7.03			
36–40 Years	41	40.85 ± 7.85			
41–45 Years	65	36.69 ± 8.93			
46 Years and above	189	39.01 ± 6.68			

* Independent sample *t*-test. ^†^ ANOVA test.

## Data Availability

The datasets can be provided by the corresponding author upon reasonable request.
